# Socioeconomic Inequalities and Mild Cognitive Impairment: Evidence From England and Japan

**DOI:** 10.1093/geroni/igab046.462

**Published:** 2021-12-17

**Authors:** Aswathikutty Gireesh, Pamela Almeida-Meza, Hashimoto Hideki, Andrew Steptoe, Dorina Cadar

**Affiliations:** 1 University College London, London, England, United Kingdom; 2 University Of Tokyo, Tokyo, Tokyo, Japan

## Abstract

Japan is the world’s fastest ageing population, with a higher prevalence of dementia than in the UK. Less clear is the role of socioeconomic inequalities in neurocognitive disorders between these countries. This study aims to assess comparatively the relationship between education, a marker of cognitive reserve, and income in relation to mild cognitive impairment (MCI) and dementia in England and Japan. We ascertained MCI using a validated algorithm based on one standard deviation below the mean on two standardised cognitive tests. Multinomial logistic regression models were used to study the associations between socioeconomic markers and MCI/dementia. The prevalence of MCI was almost twice as high among English adults compared to Japanese. Results suggest that nations are similar in overall socioeconomic inequalities of MCI/dementia, but this might differ across socioeconomic markers. Considerable variability in the health inequalities could be attributed to the country-specific socio-cultural-political factors, which remains to be further explored.

